# Modeling Planarian Regeneration: A Primer for Reverse-Engineering the Worm

**DOI:** 10.1371/journal.pcbi.1002481

**Published:** 2012-04-26

**Authors:** Daniel Lobo, Wendy S. Beane, Michael Levin

**Affiliations:** Center for Regenerative and Developmental Biology, and Department of Biology, Tufts University, Medford, Massachusetts, United States of America; National Institute of Genetics, Japan

## Abstract

A mechanistic understanding of robust self-assembly and repair capabilities of complex systems would have enormous implications for basic evolutionary developmental biology as well as for transformative applications in regenerative biomedicine and the engineering of highly fault-tolerant cybernetic systems. Molecular biologists are working to identify the pathways underlying the remarkable regenerative abilities of model species that perfectly regenerate limbs, brains, and other complex body parts. However, a profound disconnect remains between the deluge of high-resolution genetic and protein data on pathways required for regeneration, and the desired spatial, algorithmic models that show how self-monitoring and growth control arise from the synthesis of cellular activities. This barrier to progress in the understanding of morphogenetic controls may be breached by powerful techniques from the computational sciences—using non-traditional modeling approaches to reverse-engineer systems such as planaria: flatworms with a complex bodyplan and nervous system that are able to regenerate any body part after traumatic injury. Currently, the involvement of experts from outside of molecular genetics is hampered by the specialist literature of molecular developmental biology: impactful collaborations across such different fields require that review literature be available that presents the key functional capabilities of important biological model systems while abstracting away from the often irrelevant and confusing details of specific genes and proteins. To facilitate modeling efforts by computer scientists, physicists, engineers, and mathematicians, we present a different kind of review of planarian regeneration. Focusing on the main patterning properties of this system, we review what is known about the signal exchanges that occur during regenerative repair in planaria and the cellular mechanisms that are thought to underlie them. By establishing an engineering-like style for reviews of the molecular developmental biology of biomedically important model systems, significant fresh insights and quantitative computational models will be developed by new collaborations between biology and the information sciences.

Possibly the people who are trying to discover how to set up a computer to learn to play good chess, or bridge, are among those most likely to make a major contribution to the fundamental theory of evolution. — C. H. Waddington [1]

## Introduction

The ability to control the pattern formation of organs and appendages is a key aim of regenerative medicine. Transformative impact in areas such as birth defects, traumatic injury, cancer, and degenerative disease requires that we understand the molecular mechanisms that allow living beings to detect and repair damage to complex biological structures. A similar goal is pursued by engineers seeking to build resilient machines and fault-tolerant, robust systems. A medical treatment that would enable a person to regenerate a completely new head, or a robotic system that could automatically recover its proper structure and function after losing more than 99% of its constitutive parts, is still only a dream. However, there does exist a natural system capable of performing these amazing feats: the planaria.

Planarians are nonparasitic flatworms that have bilateral symmetry, a true brain driving a complex behavioral repertoire [2], and an extraordinary capacity to regenerate due to the presence of a large adult stem cell population [3]. Individual planarians are practically immortal—able to regenerate aging, as well as severely damaged or lost, tissues [4]. A trunk fragment cut from the middle of an adult planarian will regenerate into a whole worm, always growing a new head and new tail in the same orientation as the original worm. As little as 1/279th of a planarian [5], or a fragment with as few as 10,000 cells [6], can regenerate into a new worm within 1–2 weeks. Planaria are a popular model for molecular-genetic and biophysical dissection of pathways that underlie regenerative patterning [4], [7], [8], having more genes in common with humans than with the fruit fly *Drosophila*. A mechanistic understanding of the communication and control networks that maintain complex shape against radical perturbations will revolutionize our ability to regulate stem cell behavior in the context of the host organism. Thus, reverse-engineering the remarkable system that is planarian regeneration would have profound impacts on regenerative medicine, bioengineering, synthetic biology, and robotics.

Regeneration in planarians involves a truly complex interaction of several systems at the organismal level. After an injury, the stem cells in the worm proliferate and migrate to form a protective mass of new cells (blastema) at the wound site. This cell proliferation is tightly coordinated with the selective destruction of some old cells (apoptosis), effectively remodeling both the new and old tissues to recreate exactly those regions and organs the worm is missing, adjust the proportions of the remaining regions and organs to the new smaller worm size, and maintain the original patterning orientation of the worm with the new tissues. These complex interactions are controlled by a diverse set of signals, including molecular pathways, gap junctional communication, ion fluxes, and nervous system signals. Although essential for regeneration, the mechanisms by which these signals integrate to maintain and restore the correct geometry of the animal are still not well understood.

After more than 100 years of research, no single model has been proposed that explains comprehensively the mechanisms of all the known components of planarian regeneration; the majority of current models are descriptive in nature and limited to only one or two observed properties [9]–[12]. Current research efforts capitalize on molecular and cell biology techniques to produce an ever-increasing set of detailed data on genetic components that are necessary for normal regeneration [13]. However, making use of such information for biomedical or engineering purposes requires the integration of protein or gene networks into constructive models that are sufficient to predict and explain geometry of tissues and organ systems, and reveal what changes must be made in specific signals to drive necessary alterations of tissue topology. If we hope to understand and tame powerful regenerative mechanisms, we will need to develop algorithmic models that are consistent with the existing experimental datasets but also bridge the gap between functional genetic data and self-assembly of three-dimensional shape and dynamic morphostasis. Algorithmic (also called mechanistic or computational) models, in contrast to descriptive ones, explain precisely at every step what information a system needs and what logical steps should be performed, i.e., what algorithm governs the observed processes [14], [15] (for excellent introductions to biological pattern formation modeling see [16]–[19]). Unlike bare gene or protein networks, such models are constructive in the sense that they make explicit the events that need to occur to create a specific shape. Only a handful of algorithmic models have been proposed over the years to explain regeneration in planarians [20]–[23] (see “Existing Models” section below and Supplemental Text S1), and none of them successfully integrate more than one or two key features of regeneration.

There is a gap between the success of high-resolution genetic analysis and the needed level of insight into systems-level mechanisms that enable adaptive control of pattern formation. A fresh set of ideas may be helpful, from areas of science that have developed techniques for reverse-engineering complex systems, utilizing analytical methods and types of models that are distinct from those familiar to most cell biologists today. Construction of *in silico* implementations is especially crucial; for any but the most trivial set of relationships among subunits, running a simulation on a computer is the only way to determine the predictions of a given system of rules, ascertain the model's quality of fit to the known data, derive testable predictions for driving real experiments, and determine which manipulations can give rise to desired patterning outcomes.

To facilitate the application of engineering and information sciences to this fascinating problem [24]–[26], experts outside of molecular and developmental biology need to become aware of the basic capabilities of the planarian model system and the current state of knowledge about the control mechanisms involved. The first reviews to highlight the remarkable regenerative capacity of planaria were mainly descriptive collections reporting on various cutting experiments [27], [28]. Later, functional experiments were also described, including starvation, transplantations, irradiation, and pharmacological exposures [29], [30]. Given the revolution in available molecular methods, the most recent reviews have superbly summarized the genetics of regeneration [4], [10], [31]–[34], detailing the growing number of gene products whose experimental inhibition results in various kinds of regenerative failures. Unfortunately, these reviews are largely unusable by computer scientists or engineers, as the molecular details of pathways and protein–protein interactions obscure the main features and control functions to be modeled.

In this review, we hope to close the gap between regenerative biology and the fields of mathematics, computer science, and engineering and lower the barrier for experts from the information and systems engineering sciences to apply their knowledge to unraveling the mechanisms of large-scale regeneration. Here we provide an overview of the planarian regeneration system, explain what is known about the signaling mechanisms, summarize the proposed partial models in the literature, and frame the specific issues that must be addressed to bring the power of interdisciplinary investigation to fruition. Our goal is to present the basic features of this system from an engineering perspective to facilitate modeling approaches [35]–[38]. If the modeling and engineering communities can be engaged to produce algorithmic models that can accurately explain the regeneration process, the application of biologically inspired computational ideas will feed back into biology and aid our understanding of complex biological systems [39]. Conversely, the insights gained from the construction and application of these regenerative models will equally benefit computer science, artificial life, robotics, and many areas of engineering. Moreover, we hope this review will have the broader impact of establishing a precedent for much-needed different kinds of reviews that lower the barrier for true interdisciplinary cross-fertilization. Planaria constitute an excellent test case with which to explore this type of approach.

## The Building Blocks for Modeling Planaria

### Basic Anatomy and Physiology

**Figure 1:**
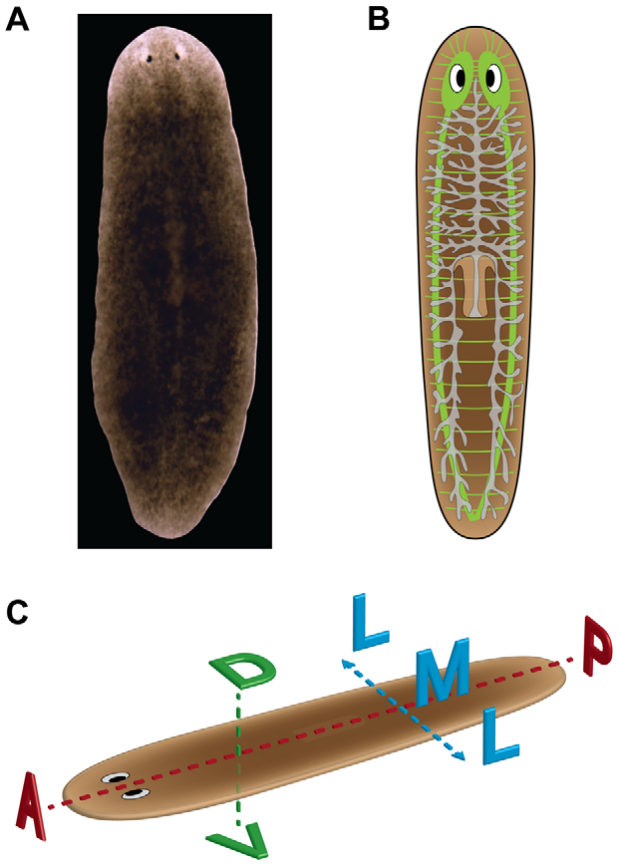
Planarian anatomy and body axes. (**A**) Dorsal side of the planarian *Schmidtea mediterranea*. (**B**) Planarian diagram showing the brain lobes, nerve cords, and secondary nerves (green); the two eyes (black and white); the gastrovascular tract (gray); and the pharynx (light brown). (**C**) The three main axes of the planarian anatomy: anterior-posterior (AP), dorsal-ventral (DV), and medial-lateral (ML).

Several species are used for research; Figure 1 summarizes the basic anatomy of *Schmidtea mediterranea* planaria and outlines their major anatomical axes. Planaria possess an intestine (gastrovascular tract), a body-wall musculature, a well-differentiated nervous system (including brain) with most of the same neurotransmitters as humans, three tissue layers (endoderm, ectoderm, and mesoderm), and bilateral symmetry [3]. The gastrovascular tract consists of a highly branched gut spread throughout the entire body, with a single ventral opening from which a long muscular tube (the pharynx) both takes in food and expels wastes [40]. The central nervous system is comprised of a bi-lobed cephalic ganglia (the brain) connected to two ventral nerve cords that run longitudinally throughout the animal and fuse in the tail [41]. Planarians possess a diverse set of sensory receptors that can detect light [42], [43], chemical gradients [44], [45], vibration [46], electric fields [47], magnetic fields [48], [49], and even weak γ–radiation [50]. At least 20–30 different types of planarian cells have been described [51], [52], including broadly distributed pluripotent adult stem cells called neoblasts that constitute 20%–30% of the total planarian cell population [53]. Neoblasts are the only proliferative cells in the body, with the ability to differentiate into any other planarian cell type, and are a key component of the planarian's ability to regenerate [54].

### Regeneration Primer

Planarians have the remarkable ability to regenerate an entire worm from a fragment that may lack any brain, central nerve cords, or pharynx. Regeneration is completed through (1) closure of the wound within the first 30–45 min, (2) formation of a mass of new cells (called the blastema) at the injury site, which is visible by 48–72 h, and (3) re-patterning of both the old and the new tissues over the next 1–2 weeks. These processes together restore the normal morphology of the worm.

Wound closure is facilitated by muscle contraction [55], but the molecular trigger for this reaction is unknown. However, migration of planarian epithelial cells to the wound site is known to be an essential component for wound closure [56], as is the juxtaposition of dorsal and ventral tissues [57]. Wound closure is followed by an initial body-wide peak of cell division (mitosis) from the neoblasts within 6 h after injury; if tissue was lost, this is accompanied by the migration of neoblasts towards the wound by 18 h and a second, local mitotic peak at the injury site around 48–72 h after injury to promote the formation of the blastema [53]. Conversely, these proliferative spikes are counterbalanced by an initial local increase of cell death (apoptosis) at the wound site within 1–4 h after injury, followed by a second, systemic apoptotic increase throughout the body that peaks at about 72 h after injury as old tissues are remodeled [58]. Thus, normal morphology is restored by a tightly regulated combination of new tissue growth and selective loss of old tissues, producing a new worm that has all its parts in the correct proportion for its now smaller size [59].

Planarians also use this extraordinary regenerative ability to reproduce asexually. Through the process of transverse fissioning, planarians anchor their tails and essentially pull themselves apart, resulting in two fragments (one head and one tail) that will regenerate into two genetically identical worms [3].

### Signaling Mechanisms

Regeneration in planarians has been shown to be the result of carefully orchestrated communication both within the blastema and between the newly regenerating tissues and the old existing ones [8], [60], [61]. However, the exact nature of this communication is not well understood and is the focus of a majority of planarian experiments, which together support four main types of signaling mechanisms: cell signaling pathways, gap junctional communication, ion fluxes, and nervous system signals.

In planarians, as in other multi-cellular organisms, classical cell signaling pathways exist that allow cells to communicate with each other [10]. Diffusing chemical factors act as messenger molecules, triggering a specific cascade of events either in the same cell or, more often, in neighboring cells. Typically, developmental signaling pathways involve the secretion of a molecule (morphogen) from one cell that binds to its specific receptor on a second cell (which can be located quite distant from the originating cell). Sometimes, the same morphogen is dispersed throughout the animal in a concentration gradient, where different concentration ranges of the same morphogen produce different outcomes. This type of signaling is widespread and used in both short-range and long-range cell communication for basic cellular activities such as rearranging the cytoskeleton, changing gene expression, and global tissue patterning during embryogenesis.

Alternatively, cells can communicate with their immediate neighbors through the direct exchange of cytoplasm in a process known as gap junctional communication (GJC) [62]. Gap junctions are membrane channels that dynamically allow for the direct transmission of small molecules and ions between cells; gap junctions are passive channels that can control the amount and type of small molecules that pass through. Hence, GJC enables regulated quick bursts of communication, permitting synchronization among nearby cells, while inhibition of GJC can create regions of isolation often needed for morphogenesis [62]. The movement of charged substances through GJC-connected cells can be driven by electrophoretic forces [63], [64]. Furthermore, voltage gradients can be transmitted and altered through gap junctions, such that cells are able to sense the membrane potential of neighboring cells. In planaria, about a dozen gap junction genes (the *innexin* family) have been found [65], [66], and various combinations create junctions that selectively allow for different degrees of communication among cells, such as that between neoblasts and their differentiated neighbors [66].

While ion flux is more commonly associated with nerve conduction, all cells generate and receive bioelectrical signals through channels and pumps within their membranes, producing ion currents which, in turn, create voltage gradients [67]. Recent experiments have begun to identify these long-term, steady-state membrane voltage gradients and ion flows, the genetic networks that regulate them, and the mechanisms that transduce electrical signals into changes in patterning and morphogenesis [68]. Thus, bioelectric signals (often transmitted by GJC) and signaling pathways interact, and together they regulate regenerative outgrowth, cell proliferation, differentiation, and migration [69]. Recent work has begun to elucidate the bioelectric signaling that carries patterning information during planarian regeneration, including membrane voltage gradients, and fluxes of hydrogen, potassium, and calcium [70]–[72]. Investigations in multiple model species have shown that several cellular mechanisms can transduce such electrical signals into intracellular responses, including: control of Ca^2+^ flux and calcium-mediated genetic regulation [73], phosphorylation of key regulatory enzymes induced by electric fields [74], subcellular translocation of transcription factors due to depolarization [75], modulation of voltage-sensitive transporters, redistribution of membrane receptors, electrophoresis of signaling molecules such as morphogens, and activation of signals by voltage-induced conformational changes in membrane proteins [67].

Finally, the nervous system itself may mediate instructive signaling during planarian regeneration [30]. Recently, it has been shown that the planarian's ventral nerve cords have the capacity to transmit long-range information to a wound site regarding the presence or absence of anterior tissues in a fragment following amputation [8]. It is clear that a comprehensive picture of regenerative regulation will include the integration and coordination of all of these overlapping signaling mechanisms.

## Planarian Experiments: The Current Dataset

A large amount of functional data has been generated over the last century that remains to be integrated into models that explain the generation and repair of complex structures during planarian regeneration. Before the emergence of modern molecular tools, the most basic of regeneration experiments involved cutting assays, in which a section of the planarian was amputated. Almost all conceivable types of amputations are documented in the classical literature [30], with most of them resulting in the regeneration of a complete worm. The majority of current planarian experiments consist of inhibiting or silencing the expression of a protein encoded by a specific gene through pharmacology or RNA interference (RNAi) and using the results to determine the regeneration mechanism in which that gene is involved [76], [77].

**Figure 2:**
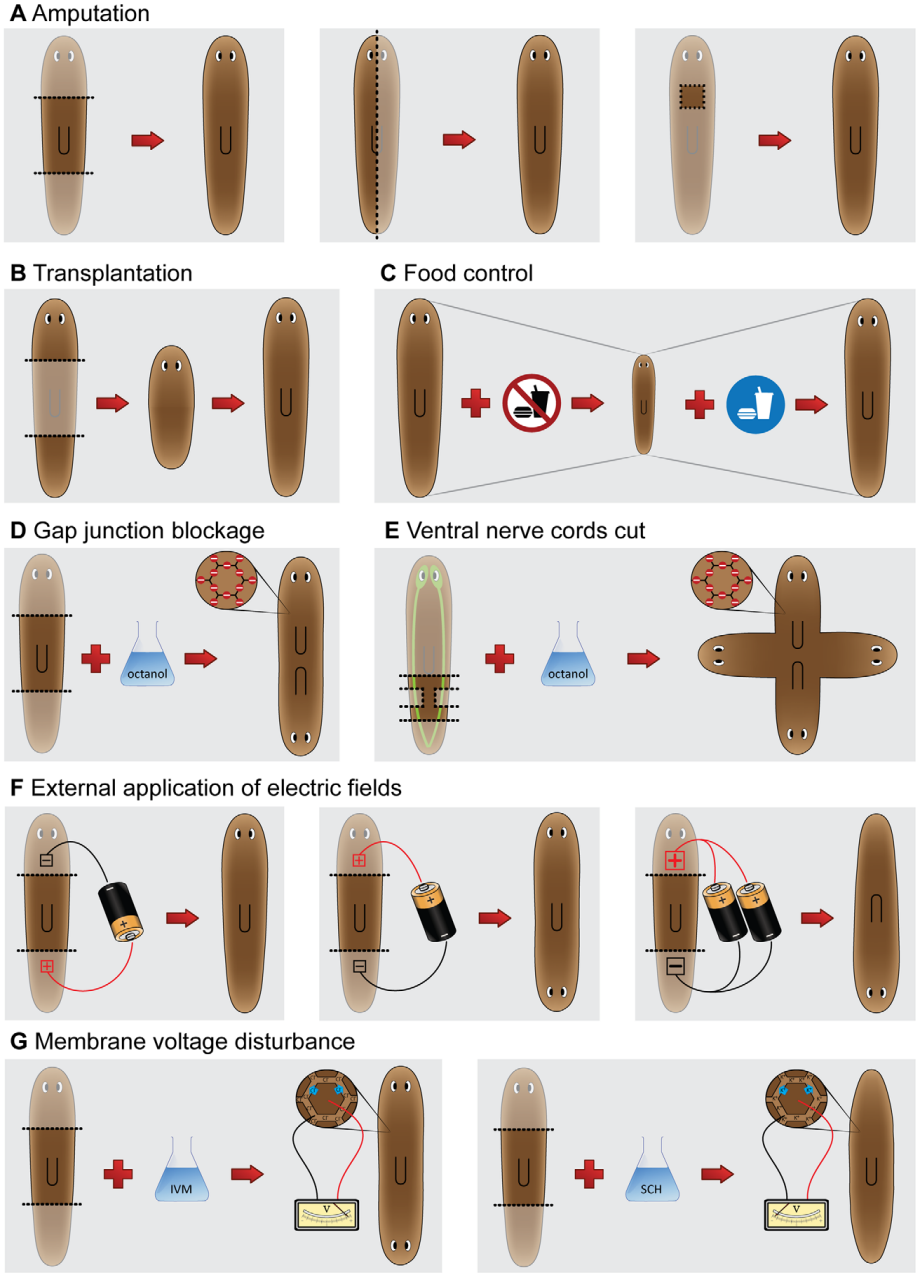
Diagrams of the main planarian regeneration experiments found in the literature. (**A**) Cutting experiments amputate part of the planarian body (shadowed); normally, a complete regenerated worm results within 1–2 weeks. (**B**) Transplantation of diverse parts also regenerates into a complete worm. (**C**) Planarians degrowth when starved; they restore their original size upon feeding. (**D**) Octanol blocks gap junction communication between the worm cells; a trunk fragment treated with octanol regenerates into a double-headed worm. (**E**) A post-pharyngeal fragment treated with octanol and with the nerve cords partially amputated regenerates into a quadruple-headed worm. (**F**) An external electric field applied to a trunk fragment disturbs AP polarity during regeneration when the anode is located in the head wound; low-intensity currents cause double-headed worms, whereas high-intensity currents cause reversed-polarity worms. (**G**) The drugs *ivermectin* (*IVM*) and *SCH-28080* (*SCH*) disturb the ion pumps in the worm cells, altering their membrane voltage. *IVM* causes cell depolarization (more positive) and trunk fragments to regenerate into double-headed worms; *SCH* causes cell hyperpolarization (more negative) and trunk fragments to regenerate into worms with no heads.

Any useful model of planarian regeneration must exhibit the behavior observed in these functional experiments. To facilitate efforts at constructing such models, in this section we highlight a selection of the main types of experiments found in the literature (Figure 2). For convenience, we organized the experiments into several broad categories of the kind of regenerative questions these experiments attempt to answer. This is not to suggest that the mechanisms in planarians are independent or modularized in this way. Indeed, computational models that do not have any pre-determined organizational bias are more likely to uncover significant, novel regenerative mechanisms.

### How Regeneration Is Initiated

Following injury, a cascade of signaling events is triggered that initiates the regeneration process. Experiments using markers for both neoblasts and their mitotic activity have provided evidence for the existence of two different signaling events, which distinguish between simple wounding (resulting in wound healing alone) and the loss of tissue (requiring regeneration) [9]. After wounding, an increase in neoblast mitoses occurs throughout the animal; however, only tissue loss results in neoblast migration to the wound site and a second mitotic peak at the wound resulting in blastema formation [9]. The signal that causes neoblast cells to stop proliferating and start differentiating within the blastema requires the activation of the extracellular signal-related kinase (ERK), since pharmacological experiments blocking ERK result in blastema formation without neoblast differentiation [78]. Finally, c-Jun N-terminal kinase (JNK) signaling is required for blastema formation [79], while epidermal growth factor (EGF) receptors have been shown to regulate the differentiation of several cell types during regeneration [80].

### How Polarity Is Established

**Figure 3:**
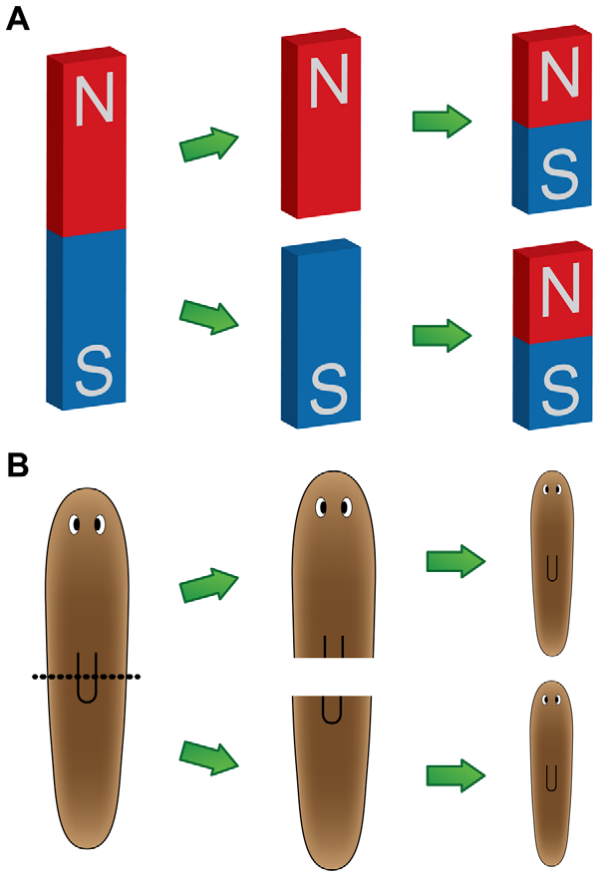
Planaria restore their AP polarity similarly to bar magnets. (**A**) A bar magnet restores the original polarity after being cut transversally. (**B**) Similarly, after bisecting a worm, polarity is restored correctly in each fragment. Note that even though cells on either side of the amputation plane were direct neighbors before the cut, the ones facing posterior make a completely different structure (a tail) than the ones facing anterior (a head). The drastically different fates of cells with essentially the same positional information suggest models based on non-local control of orientation [8].

Polarity refers to an asymmetric distribution of a particular property [81]. Planarians have several polarities along their body axes, with anterior-posterior (AP, or head versus tail polarity) and dorsal-ventral (DV, or top versus bottom polarity) being the most prominent in the literature. Remarkably, this polarity is somehow maintained even when anatomical cues, such as the brain and pharynx, are removed. For instance, a worm trunk fragment generated by removing both the head and tail will always re-grow its head in the same orientation as the original worm (never producing a head in the direction of the original tail), thus maintaining its AP polarity (Figure 3).

Although regeneration usually results in the formation of a whole worm regardless of the injury that initiated it (Figure 2A), there are exceptions. Very thin cut fragments sometimes regenerate with symmetrical double heads, although never double tails [5]. Also, really long worms sometime spontaneously produce double heads after fissioning [82]. Such failures represent excellent opportunities for dissecting the molecular mechanisms that drive planarian regeneration as well as guiding the choice of signaling modes used for modeling this system.

Several signaling pathways are involved in the regulation of planarian AP polarity. The Wnt/β-catenin pathway, comprising a large number of regulatory proteins and signaling molecules, is essential for the formation of the primary body axis in most animals [83]. This pathway is known to be necessary for posterior polarity (tail formation) during regeneration in planarians, and its perturbation causes head regeneration at every wound regardless of the original polarity [84], [85]. The Wnt/β-catenin pathway in planarians is in turn regulated by the hedgehog (Hh) pathway, which also is required for posterior polarity and when blocked similarly results in only head regeneration [86], [87]. Conversely, both the β-catenin destruction complex member adenomatous polyposis coli (APC) and the Wnt/β-catenin pathway inhibitor *notum* are required for anterior polarity (head formation), and their loss leads to the regeneration of tails only [88], [89]. Loss of *axin* genes (other negative regulators of the Wnt/β-catenin pathway), or the exogenous administration of retinoic acid (a small molecule that is also important for vertebrate AP patterning), are also required for planarian anterior regeneration [90], [91].

Bioelectric signals also regulate planarian AP polarity. A series of classical experiments showed that applying external electric fields to regenerating trunk fragments can result in AP polarity reversals [92], [93] (Figure 2F). Regeneration proceeded normally when the anterior cut faced the cathode (negative), while double-headed worms were produced when the anterior cut faced the anode (positive). With the application of higher current densities, there occurred the regeneration of morphologically normal worms but whose AP polarity was completely reversed compared to the original fragment. More recently, pharmacological experiments targeting endogenous ion channels and pumps in worm cells revealed a membrane voltage signaling pathway that is required early for the regeneration of heads [70]. Membrane depolarization of the blastema (endogenously regulated by hydrogen and potassium flux through membrane ion translocator proteins) is required for head regeneration; the data suggest that a voltage-mediated influx of calcium into the blastema triggers anterior gene expression [70], [72]. In fact, forced depolarization of a blastema is sufficient to cause head regeneration even at posterior wounds (Figure 2G) [70].

There is evidence that the organization of the cellular cytoskeleton may also play a role in AP polarity, as inhibition of microtubule organization (using the inhibitor colchicine) induces bipolar head phenotypes in regenerating planarian trunk fragments [94], [95]. Gap junctional communication has also been demonstrated to be necessary for AP polarity in planaria. Both pharmacologically (through exposure to long-chain alcohols such as octanol, which inhibit GJC) and genetically (using RNAi to abrogate expression of planarian *innexin* gap junction proteins), it has been shown that isolating cells from gap junction-based communication with other regions of the worm leads to inappropriate generation of secondary heads [8], [65], [66] (Figure 2D). The ventral nerve cords seem to similarly transmit information along the planarian AP axis during regeneration [8] (Figure 2E). Thus, like Wnt/β-catenin and Hh signaling, GJC- and neural-mediated signaling appear to be equally necessary for blastema cells to determine the needed identity of the structures they assemble. It is tempting to hypothesize that both of these systems underlie the long-range information exchange between existing tissues and sites of active morphogenesis that is needed for the regeneration of the needed structures, and only those structures.

The establishment and maintenance of the DV axis during regeneration in planarians is regulated by the secreted bone morphogenetic protein (BMP) pathway. While in vertebrates BMP is expressed on the ventral side, in invertebrates BMP is expressed dorsally [96], [97]. In planaria, BMP signaling drives dorsal fates, while BMP inhibition results in ventralized planarians [98]–[100]. Similarly, silencing of *noggin* genes, which are inhibitors of BMP signaling, results in dorsalized planarians [101]. Also important for DV polarity is the antidorsalizing morphogenetic protein (ADMP), which promotes, but also is inhibited by, BMP; they are thought to create a BMP/ADMP regulatory circuit to control DV polarity [11]. Thus ADMP ensures that BMP continues to be expressed, while BMP expression subsequently turns off ADMP expression, producing two non-overlapping domains of ADMP (ventral) and BMP (dorsal) that establish the DV axis.

Animals with bilateral symmetry, including planarians, also have medial-lateral (ML) polarity. A gene from the *slit* family is expressed along the planarian midline; when knocked down, ML polarity collapses and gives rise to phenotypes such as the regeneration of a single, cyclopic eye [102]. The protein Wnt5 is secreted from lateral cells and restricts the expression of the *slit* gene to the midline; loss of Wnt5 results in the expression of *slit* beyond the midline, disrupting ML polarity and leading to regeneration phenotypes such as multiple, ectopic pharynxes [103], [104]. Interestingly, as in vertebrates, in which the dorsal-ventral and left-right axes are linked, in planaria DV polarity also appears to affect ML polarity. For instance, as with Wnt5, loss of ADMP (which regulates BMP signaling) also results in the lateral expansion of *slit* expression [11]. Finally, it should be noted that planaria are not quite symmetric about the left-right axis: although the extent of consistent asymmetry and its underlying mechanisms are completely unknown, the left eye has a significantly better capacity to regenerate under pharmacological perturbation of eye-relevant ion currents following head amputation [71].

### How Tissue Identity Is Determined

A central component of most planarian regeneration studies is the question of how cell type and tissue identity are specified in each location [105]. Although the ultimate goal is to understand how every planarian tissue and cell type is regenerated and maintained, here we concentrate on the most widely studied tissue types into which neoblasts differentiate [106]. Such studies are aided by the fact that the stem cell population can be selectively killed by irradiation, which prevents planarians from regenerating [30]. This method has been used to show that a single transplanted neoblast can rescue the regenerative capacity of irradiated planarians, as well as induce the production of gonads in asexual hosts [54]. Irradiation experiments have also been used to elucidate molecular identifiers of neoblasts. The *piwi* family of regulatory genes, known to be essential for maintaining stem cell populations by preventing cell differentiation, are expressed solely in neoblasts and are required for regeneration [107]–[109]. Biological markers that distinguish both early and late neoblast prodigy (descendants that will differentiate into tissue-specific cells) have also been identified [110].

GJC is also important for neoblast survival, as inhibition of the gap junction protein innexin-11 results in a loss of both *piwi*-positive cells and of the ability to regenerate [66]. Additionally, the phosphatase and *tensin* homolog (*PTEN*) protein (a known tumor suppressor) and its respective pathway have been shown to regulate neoblast activity in planarians. Disruption of planarian *PTEN* signaling results in both the hyperproliferation of neoblasts and the appearance of abnormal outgrowths reminiscent of tumors [111]. In mammals, loss of PTEN activates the target of rapamycin (*TOR*) pathway, resulting in tumor formation [112]. Pharmacological inhibition of TOR (with rapamycin) in planarians rescues loss of PTEN, preventing abnormal outgrowths and restoring regenerative ability [111]. Understanding the factors that link cell-level regulation of neoblast proliferation and differentiation to system-level needs such as polarity and patterning is a crucial next step for the field.

The planarian nervous system contains a full set of vertebrate neurotransmitter homologs, possessing among others dopaminergic, serotonergic, cholinergic, and GABAergic neurons [113]–[116]. Neural connectivity is mediated in part by the *disheveled* family of proteins, a *Wnt* signaling pathway member, most likely *Wnt5* signaling [117]. Interestingly, several of the ML polarity regulators are also involved in the regulation of nervous tissue organization and when inhibited result in neural patterning defects in which nerves collapse towards the midline (*slit* inhibition [102]) or, conversely, displace laterally (Wnt5 inhibition [117]). The fibroblast growth factor (FGF) pathway, important in vertebrate neural formation and patterning, also participates in planarian brain regeneration. Loss of function of the gene *nou-darake*, a component of the FGF pathway specifically expressed in the head region, leads to the expansion of the brain through the body [118]. Finally, the netrin family of axon guidance proteins is also required in planarians for the regeneration and maintenance of neural patterning; when the netrin receptor is inhibited the overall organization of the nervous system is lost, disrupting the relationship between the brain and VNCs [119].

Graft transplantation experiments have historically been used to probe the functional identity of different regions and tissues in planaria and have provided important information about the types of signaling that occur between different tissues (Figure 2B). For example, when a small piece is cut out, flipped along the DV axis, and grafted back into its original location in the worm, a cup-shaped projection is formed on the boundary between the host and the graft; for anterior grafts this projection will develop a head-like morphology, while posterior projections will appear tail-like [21]. This suggests that the juxtaposition of dorsal and ventral tissues is a cue that signals the regenerative process. In contrast, if two dorsal halves are grafted together, no regeneration occurs [22]. Such results have driven many models in vertebrate systems in which growth is dictated by juxtaposition of regions with distinct “positional information values” [120]. Similarly, if the pharyngeal region is removed and the remaining head and tail fragments are then joined together, a new pharynx regenerates between them [121]; however, when two fragments from the same region of the worm are grafted together, no regeneration is observed [21]. Finally, if a small fragment of the head is grafted into the tail region, a new head and pharynx will appear with reversed polarity from the original AP axis; conversely, if a tail piece is grafted anterior to the pharynx, a second pharynx will form between the head and the transplanted tissue, again with reversed AP polarity [60], [61]. These results clearly indicate that, instead of adopting the identity of the host tissue, a grafted piece can signal the host to re-pattern and change tissue identities.

During everyday life, planarian cells are continuously replaced by the differentiating progeny of the neoblast population in a process known as morphostasis [4]. The mechanism is not well understood, although it is known that mitogen-activated protein kinase (MAPK) signaling is required for neoblasts to stop proliferation and undergo differentiation [78]. This homeostatic process of cell turnover, which constantly renews all cells without changing tissue size or proportion [55], [122], is common among organisms. However, planaria exhibit a unique remodeling ability that enables them to dynamically scale their body in relation to the available cell number. Upon starvation, planaria use their own cells for energy, reducing their body size in a phenomenon known as degrowth [3]; this is reversible, and growth resumes upon feeding (Figure 2C). The worm's total cell number changes linearly with respect to body length and time, while keeping the proportion of all cell types constant. This requires active remodeling of existing tissues to scale the structure in a process known as morphallaxis and is quantitatively described by an allometric equation [52], [123]. The mechanisms regulating this exquisite rearrangement are not well understood, but it is accomplished in part through coordinated cell death and neoblast proliferation [58], [124].

This brief overview of some of the questions that the planarian regeneration field has investigated, both historically and currently, highlights the increasing wealth of information that continues to be generated and must be integrated into any comprehensive model of planarian regeneration. Even within a narrow area (e.g., AP polarity) the field lacks a truly cohesive model that explains the system-level (patterning) data in terms of the behavior of lower-level (cell pathway) components.

## Existing Models of Planarian Regeneration

Most modern planarian regeneration studies result in a gene regulatory network or protein interaction pathway. However, these networks largely do not constrain shape or shape regulation, and such pathways are compatible with a very broad range of morphologies. A few researchers have proposed algorithmic models, which precisely define the steps that cells (or tissues) must take to assemble or repair a given morphology. In specifying the information needed to make decisions, such models could be fleshed out in molecular terms to provide testable hypotheses about the mechanisms underlying information exchange, computation, and links to the ultimate execution of morphogenesis via cellular effectors. Due to space constraints, the basic features (assumptions and outputs) are given in Supplemental Text S1.

Many algorithmic models propose the existence of a coordinate system that facilitates the patterning of the organism. The positional information model [125] is based on diffusible substances that create concentration gradients; depending on the specific concentration of such substances at different locations, different cell programs are triggered [126]. The serial threshold model [20] combines positional information and cell migration to explain how planarians can restore AP polarity. Reaction-diffusion models [127] are based on diffusible substances that react with each other; they can generate most of the patterns found in biology [81] and explain regeneration of polarity in planaria [128]. Other algorithmic models proposed for planarian regeneration are based on bioelectrical signals [23], dorsal-ventral interactions [21], and the intercalation of missing regions [129].

However, none of the proposed models in the literature are able to explain more than one or two facets of planarian regeneration. There is a tremendous opportunity to integrate existing knowledge of pathways with the anatomical and physiological data to produce novel models of the worm as a system that senses damage [130], carries out the distributed processing needed to restore a target state, and then ceases growth. The major outstanding questions that a complete algorithmic model should be able to explain are summarized in Box 1, as a challenge to modelers from the computer programming, engineering, physics, mathematics, complex systems science, and artificial life communities.

**Box 1. Key Functional Questions Yet to Be Solved**

**Detection of missing tissues:** What is the signaling mechanism that triggers the regeneration of only the exact missing regions, structures, and organs?

**Growing boundaries delimitation:** What signals trigger growth and cause remodeling to cease when regeneration is completed?

**Plasticity of organism size:** How are planarians able to remodel existing tissues to maintain the correct internal organ proportions as size changes due to available cell number?

**Neoblast migration:** What are the driving forces that permit neoblasts to migrate in the direction of the wound to initiate blastema formation and how are positional values encoded (and to what spatial resolution)?

**Specification of cell types:** How are different cell types generated from the same neoblast population, and how are anatomical regions of different size (head vs. eye vs. photoreceptor cell) specified spatially?

**Organization of cells into organs:** Once cells are committed to a given cell type, what is the process that orchestrates their development into specific organs?

**Small vs. large fragments paradox:** Experimentally suppressing essential mechanisms (such as anterior-posterior positional gradients) in large fragments (for instance middle-third trunk fragments), disturbs normal regenerative patterning; so why are small pieces cut from a worm often still able to regenerate normally, despite such loss-of-function treatments, when they have suffered relatively more damage?

**Specification of target morphology:** What is the mechanism (whether directly encoded or an emergent property of the remaining tissue) that specifies target morphology during regeneration?

**Figure 4:**
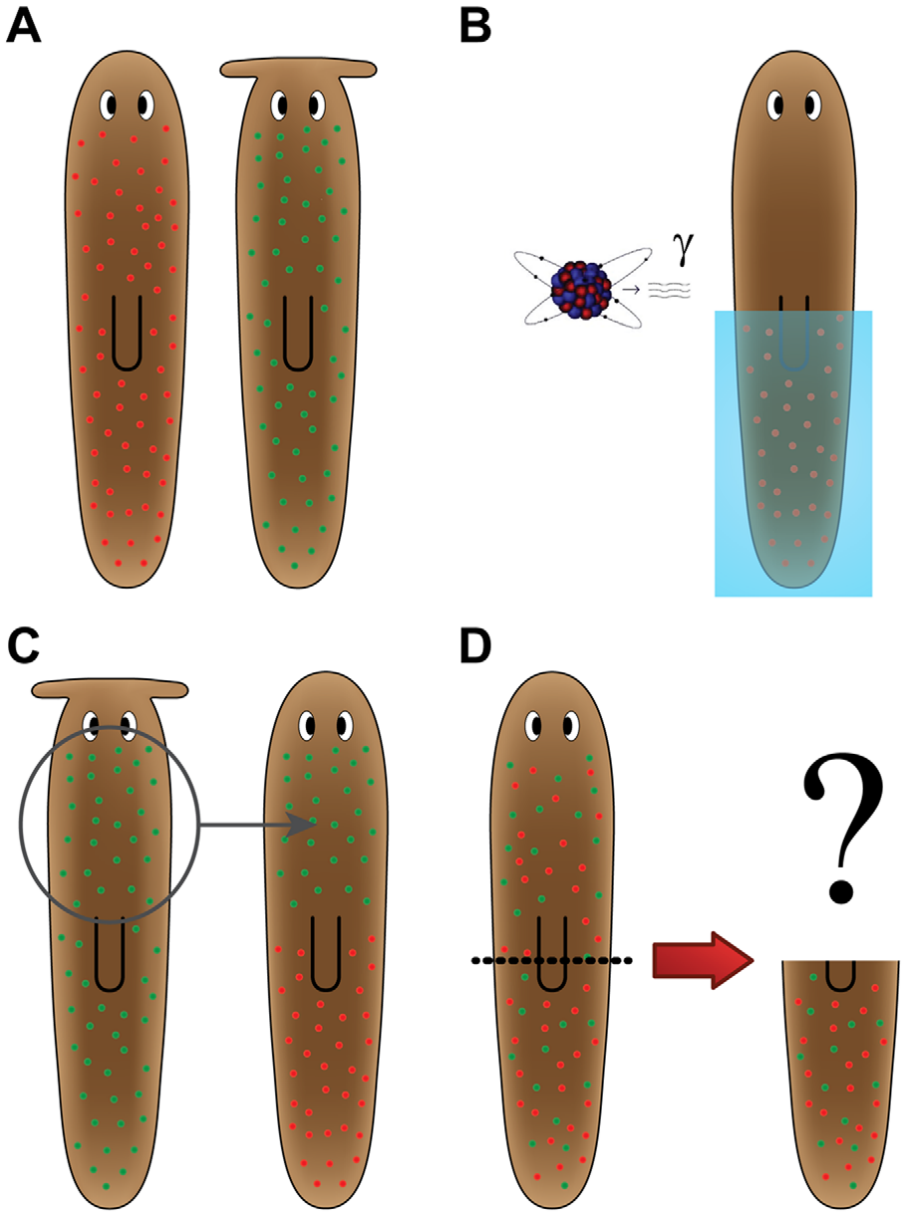
Hypothetical experiment illustrating the concept of target morphology. (**A**) *S. mediterranea* (left, rounded head) and *P. felina* (right, hammerhead) are planarian species with different head morphology. (**B**) Half the neoblasts of the rounded-head worm are killed by using irradiation and a lead shield. (**C**) Half the neoblasts of the hammerhead worm are transplanted to the rounded-head worm. (**D**) After the neoblasts have diffused, the head of the rounded-head worm is amputated. Without a model of how target morphology is determined, it is impossible to predict what shape will regenerate.

A key question concerns the possible existence of a direct encoding of “target morphology”. It is commonly held that shape is an emergent property of cell interactions. However, recent data suggest that at least basic AP polarity in worms may be directly stored. A transient modulation of physiological events in a worm results in a bipolar 2-headed outcome; remarkably, although this change did not affect the DNA sequence, the patterning change persists upon multiple subsequent amputations in the absence of any other perturbation. The shape to which the animal regenerates upon damage (the target morphology) has been stably altered, suggesting the possibility that the large-scale axial anatomic plan may be encoded in physiological networks and thus directly modifiable by non-genetic experimental interventions. The concept is highlighted in the following hypothetical experiment, illustrated in Figure 4. Take one planarian from each of two species with clearly different morphologies: *S. mediterranea* with a rounded head, and *P. felina* with a hammerhead. In this experiment, the neoblasts are killed off (by irradiation) in half of the *S. mediterranea* worm. Subsequently, live neoblasts from the *P. felina* worm are transplanted into the irradiated worm. If this chimeric worm is now cut, forcing it to regenerate its head, which head shape will be regenerated (round or hammer, or perhaps a hybrid, or perhaps regeneration will never cease as each set of neoblasts works to remodel the head)? Without a theory of how the organism's final morphology determines stem cell-driven processes during planarian regeneration, it is impossible to predict the outcome of this experiment. Truly successful algorithmic models, that can be used predicatively to forward the regeneration field, should be able to answer these types of questions.

## Summary and Conclusion

In addition to testable *in silico* models [131]–[134] (or indeed, hardware implementations based on swarm intelligence models [35]), there are several other areas in which information sciences can make a transformative impact on regeneration research. We suggest the urgent need for the development of a bioinformatics beyond sequence and regulatory networks—a bioinformatics of shape, including:

1. A formalization of patterning outcomes in model systems. We currently have no standard formal language in which outcomes such as 1-headed vs. 2-headed worm can be encoded for informatics approaches, and precise quantitative morphometrics need to be augmented by symbolic representations that focus on large-scale patterning changes.
2. Creation of databases where patterning phenotypes (and the associated manipulations that produced them) can be stored, queried, and mined. The field is currently limited to searching abstracts for keywords, and a new investigator in this field cannot easily do a search to find out if any existing experiment produced, for example, a worm with three eyes in a row.
3. A standardized formal modeling environment and representation format. Models arising from functional studies could be reported in the primary literature in a formal way that facilitated both their comprehension and implementation, to determine whether they indeed match the patterning behavior that was supposed to be explained by the genetic pathway being elucidated.
4. Artificial intelligence tools to augment human ingenuity in proposing testable models. We are currently inundated with functional data to the point that it is becoming increasingly difficult to come up with models that are consistent with available results. As this problem grows, human reasoning needs to be assisted by computational tools that can infer testable, algorithmic models from databases in which molecular-genetic perturbations are linked to their morphological outcomes.

Using the planarian regeneration data as a proof-of-principle test case, our lab is pursuing these directions. We hope the above primer on the flatworm system will motivate others with complementary expertise to join the molecular regeneration community in attempting to solve the puzzle of autonomous large-scale pattern formation and repair. Paradigms such as cellular automata [135]–[137], formal grammars [138]–[142], formal rules [143], [144], neural networks [145], and Boolean circuits [146], [147] are just some of the tools that remain to be brought to bear on this crucial problem at the center of developmental biology.

The planarian model organism represents an excellent opportunity for the application of the proposed model-building, formalization, and computational methods for automated model discovery. This approach can be also extended with little effort to other regenerative model organisms, such as hydra, axolotl, *Xenopus*, newt, zebrafish, and mammalian organs (e.g., liver, deer antlers, etc.). The incorporation of strategies from other fields, and the integration of truly interdisciplinary approaches with the now-accepted molecular genetics-bioinformatics-computational biology efforts will greatly facilitate fundamental insight into the general questions of how complex systems (organized on many scales of size) ascertain their shape, alter it to match functional needs, and recover from injury. With the ability to truly understand the generation and adaptive self-regulation of complex morphology will come exciting advances in evolutionary developmental biology, regenerative medicine, and synthetic biology. Moreover, the payoffs will extend far beyond biology, contributing significantly to cybernetics, computer science, dynamical control theory, robotics, and many areas of engineering that can benefit from understanding how living systems actually perform the remarkable tricks developed by millions of years of evolution.

## Supporting Information

Text S1 Previously proposed models of patterning in planarian regeneration.
(DOCX)
