# Supplementary material for: Modeling Planarian Regeneration: A Primer for Reverse-Engineering the Worm
Source: PLoS Comput Biol. 2012 Apr 26;8(4):e1002481. doi: 10.1371/journal.pcbi.1002481 (PMC3343107; doi:10.1371/journal.pcbi.1002481)
Supplement: Text S1 — Previously proposed models of patterning in planarian regeneration. (DOCX) [file pcbi.1002481.s001.docx]

1. Previously proposed models of patterning in planarian regeneration

Here we briefly review some of the existing efforts at modeling planarian patterning, to highlight the approaches that have been attempted and the benefits/limitations of each.

- 1. Gradient models of regeneration

Early studies into the regenerative capacities of different planaria species [1] gave rise to the original concept of a morphogenetic gradient [2] (for a historic background see [3]). The idea was based on the frequency of successful regenerations as correlated to the original location of each fragment along the AP axis [4]. A plot of this regeneration frequency against location on the AP axis provided a characteristic “head-frequency curve” for each species [5]. This curve could vary greatly among species: from a flat, complete regenerative capacity in *P. velata* to an inverse, bell-shape capacity (lower on the center and higher at both ends) in *P. dorotocephala* [4]. Inspired by this descriptive gradient of regenerative capacity, it was suggested that gradients were in fact the reason planaria could regenerate. More precisely, Morgan suggested that the observed polarity during regeneration was determined by the gradation of some materials or substances along the AP axis [6]. This head-to-tail gradient came to be thought of as either a head-promoting factor, highly concentrated in the anterior but low in the posterior, or conversely a head-inhibiting factor, with a low anterior concentration and a high posterior concentration [7]. Child proposed a variation of this gradient model, with the gradation arising in the rate of physiological processes in the organism [8,9]. Related to the concept of gradients is the organizer, which is broadly defined as a determiner or inductor of spatial developmental pattern [10,11]. Thus a gradient is an example of the output of an organizer, which can act as a source or a sink of such substance (Figure S1).


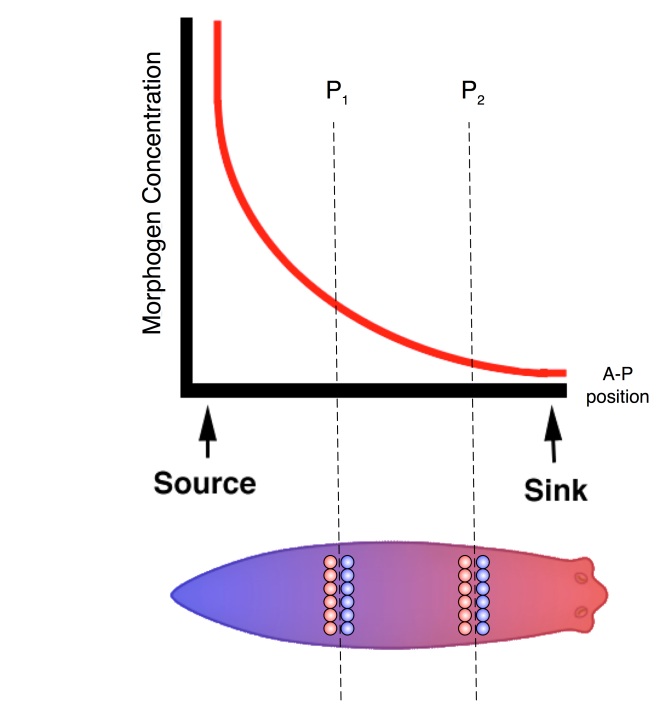


Figure S1. Gradient model explains AP polarity. A cell located in the most posterior location (source) acts as an organizer, producing a diffusing substance (morphogen). The morphogen is eliminated in the most anterior location (sink), which forms a gradient concentration over the worm. In this way, the fate of a region (tail or head) is determined by the morphogen concentration (high or low) in that area. However, the model cannot explain how cells in either side of a cut, which are located virtually in the same position and are exposed to the same morphogen concentration, have different fates. For example, after cutting the worm along P_1,_ the anterior-facing cells (red) will regenerate a head while the posterior-facing cells (blue) will regenerate a tail, being the morphogen concentration virtually identical in both sides. The same happens in other cut locations, such as P_2_.

The model of positional information extends the general concept of gradients and organizers, postulating the existence of a coordinate system that patterns the organism [12]. Specifically, a set of organizers cause a number of overlapping gradients of morphogen concentrations, with different thresholds causing different responses, and so different cell differentiation programs are induced based on different cell locations [13]. Theoretical calculations historically showed the physical plausibility of a morphogen diffusing between two regions [14]. In addition, the positional information model was complemented by the notion of positive-feedback cellular mechanisms, which explained the observed discontinuities in patterning and the persistence of the gradient after the initiating signal disappeared [15]. In summary, the current view of the gradient model consists of a fixed source of morphogen at a specific position that, through diffusion, produces gradations of morphogen concentration thus providing positional information to cells [16].

A number of specific models have been proposed to explain pattern formation by morphogenetic gradients in general or for specific signals or pathways (for reviews see [17,18]). In planaria, molecular studies have identified several morphogens that are well suited to act as the graded substances of these models [7], although no clear-cut consistent morphogen gradient has been reported. In addition, interestingly, a gradient of mitotic activity in neoblasts along the AP axis has been reported [19]. Nevertheless, gradient models alone have difficulty explaining planarian regeneration. The basic problem is that cells on either side of a cut are at virtually the same positional value but have drastically different fates (anterior-facing cells form the head while posterior-facing cells build a tail); moreover, when multiple cuts are made, the same fate (e.g., “head”) is adopted by cells that come from many different positional regions (Fig. S1, P_1_ vs. P_2_). These observations suggest the presence of long-range communication that is necessary to interpret gradient positions relative to the remaining tissue and its orientation. Likewise, the sequential patterning of a gradient predicts an equally sequential regeneration of the missing regions (i.e., the new cells generated at the wound should gradually occupy the position of the missing regions and form new structures corresponding to their location). In contrast, planarian regeneration proceeds according to an intercalation process by forming a blastema at the wound that regenerates only the most distant tissues, while the remaining missing parts are replaced by the old tissue through morphallaxis. Moreover, static morphogenetic gradients cannot account for the necessary overall remodeling that occurs as the planarian dynamically cycles between growth and degrowth. Also, mechanisms based on simple gradients require something else in the organism to setup the gradient at the correct place and time (in contrast to reaction-diffusion gradient mechanisms, see section S1.3), and hence fall short of being by themselves a complete model of pattern formation. Indeed, the system would have to be able to restore sources of the morphogen, as the gradients have to be re-established in fragments after major regions of the body are cut away. Likewise, a head organizer gradient cannot explain the continued regeneration of secondary heads after amputation of gap junction inhibitor-treated worms [20], since the ectopic heads (and any induced organizer within them) are removed by the amputation.

- 1. Serial threshold theory of regeneration

Slack proposed a unique algorithmic model of planarian regeneration to cope with the difficulty of simple gradients to explain how, after head or tail amputation, the same cells close to the wound can correctly form either a tail or a head depending on the tissues remaining [21]. This model suggested that a worm is divided into discrete territories, due to the combination of a global gradient with an ordered coded sequence of threshold switches in the cells. The most posterior and anterior territories always have the minimum and maximum possible codes respectively in the ordered threshold sequence. Two processes of regeneration are defined in the model: bifurcation and decrementation. First, bifurcation establishes the polarity of the blastema. Neoblasts migrate to the wound to initiate blastema formation and, once in their new location, reset their anatomy code (switches) as follows: if the wound tissue has a higher coding than the original code of the migrated neoblast, then the blastema tissue is set to the maximum possible code; otherwise it is set to the minimum possible code. Then, decrementation completes regeneration by orderly (from lower to higher codes) restoring the tissues that correspond to each code that should lie between the code of the wound and the new code of the blastema.

This model is able to explain the production of two-headed planarians from narrow trunk fragments, since the neoblasts that form the blastema would have the same code as the wound tissue and so behave according to a default option. The default option in planarians would be to set the maximum possible code, which would result in anterior (head) regeneration. However, the model fails to explain the classical observation that following a transverse cut, the new pharynx forms in the old tissue rather than in the blastema [22]. Therefore, the serial threshold model also has trouble accounting for morphallaxis.

- 1. Reaction-diffusion mechanisms of patterning

In a pioneering paper, Turing showed how a dynamical system based on reacting chemical substances can form self-generated, self-regulated spatial concentration patterns starting from a near-homogeneous configuration [23]. Effectively, a system of two substances that react with each other (with different diffusion rates) can explain the formation of many static and dynamic patterns found both in organic and inorganic matter. In particular, Meinhardt and Gierer have extensively studied reaction-diffusion mechanisms of biological pattern formation, discovering that a system only needs a self-enhancing reaction (local self-activation), coupled with an antagonistic reaction that acts on a longer range (long-range lateral inhibition), to be able to generate stationary concentration patterns from near-homogenously-produced substances [13,24,25]. Indeed, reaction-diffusion models can replicate most patterns found in biological systems [26]; moreover, molecular evidence in support for such models have been uncovered [16,27,28].

Reaction-diffusion mechanisms have been proposed as an explanation for the regeneration ability and maintenance of polarity in planaria. Specifically, a model consisting of two superimposed reaction-diffusion mechanisms has been proposed, formed from one head-inducer and one tail-inducer gradient with peaks at the extremes of the worm AP axis [29]. While a single reaction-diffusion mechanism could not account for different worm sizes, the dual model can. It predicts that, after head or tail amputation, the corresponding gradient inhibitor fades away and a new self-enhancing activation is triggered, which in turn restores the lost gradient peak. Triggered by this newly formed gradient, the wound regenerates either the most anterior or posterior part (as appropriate), while the old tissue is repatterned to replace the remaining missing tissues. For this to function, the two reaction-diffusion mechanisms need to be coupled (e.g., sharing the inhibitor), otherwise the gradient peaks would not necessary appear at opposite sides [26]. Moreover, to ensure the original polarity is restored in small trunk fragments, it is necessary to include a “graded competence” mechanism in the model [30]. This mechanism consists of an additional persistent global gradient that will induce the formation of the main gradient peaks at the correct ends.

It has been suggested that the planarian midline is formed by a morphogen produced by the DV border that acts as a long-range inhibitor of the local self-activated midline morphogen [30,31]. Interestingly, this is consistent with both the midline expression of slit (which maintains the ML axis) and the expression of ADMP (which helps restrict slit to the midline) at the boundary of the DV border [32,33]. Additionally, a reaction diffusion mechanism for planarian polarity was recently proposed based on the small molecules cAMP and ATP, which are widespread and diffuse via gap junctions [34]. The model is based on a single reaction-diffusion gradient, where a high concentration specifies head and a low concentration specifies tail. The model explains the polar (single head) to bipolar (double-headed) transition following GJC inhibition [19,20,35] as the result of changing from a high-low gradient to a high-low-high gradient (with a reduced wavelength) caused by the reduction of diffusion coefficient (from reduced GJC). However, none of the planarian reaction-diffusion models can explain other observed regenerative characteristics, such as the triggering of regeneration by joining dorsal and ventral tissues [36], the formation of independent AP axes in four-headed planarians [20], or the mechanistic basis for neoblast migration [30].

- 1. Bioelectric-electrophoretic model

The bioelectric-electrophoretic model is the first model of planarian regeneration that included bioelectrical signals [37]; it was proposed as an explanation of the experiments done with external electric fields that caused polarity reversals during regeneration [38,39]. The model is based on the hypothesis of a brain inhibitory substance produced continually by the brain; this substance is negatively charged and diffuses throughout the worm due to the planarian’s natural bioelectrical AP field (negative in head regions and positive in tail regions). The model explains the normal planarian regeneration as follows: after an anterior amputation, the most anterior region remaining no longer receives the charged inhibitory substance produced by the missing brain; since the bioelectrical field continues driving the substance towards the posterior region, the wound region is soon depleted; this signals the formation of a new brain as the concentration falls below the threshold required for inhibition. On the other hand, a posterior amputation does not stop the production of the charged substance since the brain is retained; hence, its high concentration at the wound inhibits head formation and a tail is regenerated. Effectively, this model can explain the external control of polarity by electrical external currents [38,40] due to the halted (double heads) or reversed (polarity reversal) movement of the charged substance. However, the model cannot be applied simultaneously for the patterning along the other axes, since only one overall bioelectrical field with a single direction can be present at the same time. Recent efforts have added molecular-genetic details to the bioelectrical controls of patterning in planaria [20,35,41,42], but a comprehensive physiological model that integrates both the voltage gradients and the long-range gap junctional-communication remains to be formulated.

- 1. Dorso-ventral interaction model

The dorsal-ventral interaction model is based on the experimental grafting studies with reversed-DV grafts, where inverted grafts regenerate with ectopic heads or tails due the interaction of dorsal and ventral tissues [36]. The model suggests that during wound closure, the ectopic interaction between dorsal and ventral tissues acts as a trigger for blastema formation. Subsequently, the blastema functions as a signaling center, inducing intercalary regeneration of the missing tissues. However, the model only works to explain the trigger mechanism (injury signal) that initiates regeneration, but does not address how the new tissue that results from the signal is appropriately patterned as either an anterior or posterior blastema.

- 1. Intercalary regeneration model

In a grafting experiment where two different regions are joined, planarian regeneration proceeds by forming exactly those structures missing between the now touching regions [43]. If one of the joining pieces is inverted, regeneration now proceeds until both sides are equivalent, even though existing structures are duplicated [21]. Consequently, a model has been suggested where each region of the worm has a positional value; regeneration occurs by recreating the missing regions of intermediate positional values which should lie between the value extremes of the two joined regions [44]. This mechanism, called intercalary regeneration, has also been found in other organisms [45]. While the planarian experiments described above involved the AP axis, intercalary regeneration has also been observed along the DV axis in planarians after transplantation of reversed DV grafts [36] and along the ML axis during ectopic grafting experiments [44,46]. However, this model is not sufficient to explain regeneration in non-grafting situations, where the blastema does not lie between two existing regions but rather occurs terminally at one end; in these experiments, a large part of the remaining tissue adopts the identity of missing regions, instead of being produced from a wound.

References

1. Egger B, Gschwentner R, Rieger R (2007) Free-living flatworms under the knife: past and present. Development genes and evolution 217: 89-104.

2. Morgan T (1901) Growth and regeneration in Planaria lugubris. Development Genes and Evolution 13: 179-212.

3. Slack JMW (1987) Morphogenetic gradients — past and present. Trends in Biochemical Sciences 12: 200-204.

4. Brøndsted HV (1955) Planarian Regeneration. Biological Reviews 30: 65-126.

5. Sivickis PB (1931) A quantitative study of regeneration along the main axis of the triclad body. Arch Zool Ital: 430-449.

6. Morgan TH (1905) “Polarity” considered as a phenomenon of gradation of materials. Journal of Experimental Zoology 2: 495-506.

7. Adell T, Cebrià F, Saló E (2010) Gradients in planarian regeneration and homeostasis. Cold Spring Harbor Perspectives in Biology 2.

8. Child CM (1911) Experimental Control of Morphogenesis in the Regulation of Planaria. Biological Bulletin 20.

9. Child CM (1941) Patterns and problems of development: The University of Chicago Press.

10. Spemann H, Mangold H (2001) Induction of embryonic primordia by implantation of organizers from a different species (Reprinted from Archiv Mikroskopische Anatomie Entwicklungsmechanik, vol 100, pg 599-638, 1924). International Journal of Developmental Biology 45: 13-38.

11. Child CM (1946) Organizers in Development and the Organizer Concept. Physiological Zoology 19.

12. Wolpert L (1969) Positional information and the spatial pattern of cellular differentiation. Journal of Theoretical Biology 25: 1-47.

13. Meinhardt H (1978) Space-dependent cell determination under the control of morphogen gradient. Journal of theoretical biology 74: 307-321.

14. Crick F (1970) Diffusion in Embryogenesis. Nature 225: 420-422.

15. Lewis J, Slack JMW, Wolpert L (1977) Thresholds in development. Journal of Theoretical Biology 65: 579-590.

16. Kondo S, Miura T (2010) Reaction-Diffusion Model as a Framework for Understanding Biological Pattern Formation. Science 329: 1616-1620.

17. Reeves GT, Muratov CB, Schüpbach T, Shvartsman SY (2006) Quantitative models of developmental pattern formation. Developmental cell 11: 289-300.

18. Wartlick O, Kicheva A, González-Gaitán M (2009) Morphogen Gradient Formation. Cold Spring Harbor Perspectives in Biology 1.

19. Oviedo N, Levin M (2007) smedinx-11 is a planarian stem cell gap junction gene required for regeneration and homeostasis. Development 134: 3121-3131.

20. Oviedo N, Morokuma J, Walentek P, Kema I, Gu MB, et al. (2010) Long-range neural and gap junction protein-mediated cues control polarity during planarian regeneration. Developmental biology 339: 188-199.

21. Slack J (1980) A serial threshold theory of regeneration. Journal of Theoretical Biology 82: 105-140.

22. Morgan T (1898) Experimental studies of the regeneration of Planaria maculata. Development Genes and Evolution 7: 364-397.

23. Turing AM (1952) The chemical basis of morphogenesis. Philosophical Transactions of the Royal Society of London Series B, Biological Sciences 237: 37-72.

24. Gierer A, Meinhardt H (1972) A theory of biological pattern formation. Kybernetik 12: 30-39.

25. Meinhardt H, Gierer A (2000) Pattern formation by local self-activation and lateral inhibition. BioEssays 22: 753-760.

26. Meinhardt H (1982) Models of Biological Pattern Formation: Academic Press.

27. Chen Y, Schier AF (2002) Lefty Proteins Are Long-Range Inhibitors of Squint-Mediated Nodal Signaling. Curr Biol 12: 2124-2128.

28. Sick S, Reinker S, Timmer J, Schlake T (2006) WNT and DKK Determine Hair Follicle Spacing Through a Reaction-Diffusion Mechanism. Science 314: 1447-1450.

29. Meinhardt H (2009) Models for the Generation and Interpretation of Gradients. Cold Spring Harbor Perspectives in Biology 1.

30. Meinhardt H (2009) Beta-catenin and axis formation in planarians. Bioessays 31: 5-9.

31. Meinhardt H (2004) Different strategies for midline formation in bilaterians. Nature Reviews Neuroscience 5: 502-510.

32. Cebrià F, Guo TX, Jopek J, Newmark PA (2007) Regeneration and maintenance of the planarian midline is regulated by a slit orthologue. Developmental Biology 307: 394-406.

33. Gaviño MA, Reddien PW (2011) A Bmp/Admp regulatory circuit controls maintenance and regeneration of dorsal-ventral polarity in planarians. Current biology : CB 21: 294-299.

34. Schiffmann Y (2011) Turing-Child field underlies spatial periodicity in Drosophila and planarians. Progress in biophysics and molecular biology 105: 258-269.

35. Nogi T, Levin M (2005) Characterization of innexin gene expression and functional roles of gap-junctional communication in planarian regeneration. Developmental Biology 287: 314-335.

36. Kato K, Orii H, Watanabe K, Agata K (1999) The role of dorsoventral interaction in the onset of planarian regeneration. Development (Cambridge, England) 126: 1031-1040.

37. Lange C, Steele V (1978) The Mechanism of Anterior-Posterior Polarity Control in Planarians. Differentiation 11: 1-12.

38. Marsh G, Beams HW (1952) Electrical control of morphogenesis in regenerating Dugesia tigrina. I. Relation of axial polarity to field strength. J Cell Comp Physiol 39: 191-213.

39. Dimmitt J, Marsh G (1952) Electrical control of morphogenesis in regenerating Dugesia tigrina. II. Potential gradient vs. current density as control factors. J Cell Comp Physiol 40: 11-23.

40. Marsh G, Beams HW (1947) Electrical Control of Growth Polarity in Regenerating Dugesia-Tigrina. Federation Proceedings 6: 163-164.

41. Oviedo N, Pearson B, Levin M, Alvarado A (2008) Planarian PTEN homologs regulate stem cells and regeneration through TOR signaling. Disease models & mechanisms 1.

42. Beane WS, Morokuma J, Adams DS, Levin M (2011) A chemical genetics approach reveals H,K-ATPase-mediated membrane voltage is required for planarian head regeneration. Chemistry &amp; biology 18: 77-89.

43. Santos FV (1929) Studies on transplantation in Planaria. Biol Bull 57: 188-197.

44. Agata K, Tanaka T, Kobayashi C, Kato K, Saitoh Y (2003) Intercalary regeneration in planarians. Developmental dynamics : an official publication of the American Association of Anatomists 226: 308-316.

45. Iten L, Bryant S (1975) The interaction between the blastema and stump in the establishment of the anterior-posterior and proximal-distal organization of the limb regenerate. Developmental Biology 44: 119-147.

46. Saito Y, Koinuma S, Watanabe K, Agata K (2003) Mediolateral intercalation in planarians revealed by grafting experiments. Developmental dynamics : an official publication of the American Association of Anatomists 226: 334-340.
